# Supplementary material for: Depletion of Small HDL Subclasses Predicts Poor Survival in Liver Cirrhosis
Source: Antioxidants (Basel). 2025 May 30;14(6):664. doi: 10.3390/antiox14060664 (PMC12190158; doi:10.3390/antiox14060664)
Supplement: Supplementary file 1 [file antioxidants-14-00664-s001.zip › antioxidants-3635023-supplementary.pdf]

Depletion of Small HDL Subclasses Predicts Poor Survival in Liver Cirrhosis

Anja Pammer [1], Tobias Madl [2,3], Hansjörg Habisch [3,4], Jakob Kerbl-Knapp [4], Florian Rainer [5], Vanessa Stadlbauer [5], Angela Horvath [5,6], Philipp Douschan [5,7], Rudolf E. Stauber [5] and Gunther Marsche [1,3,*]

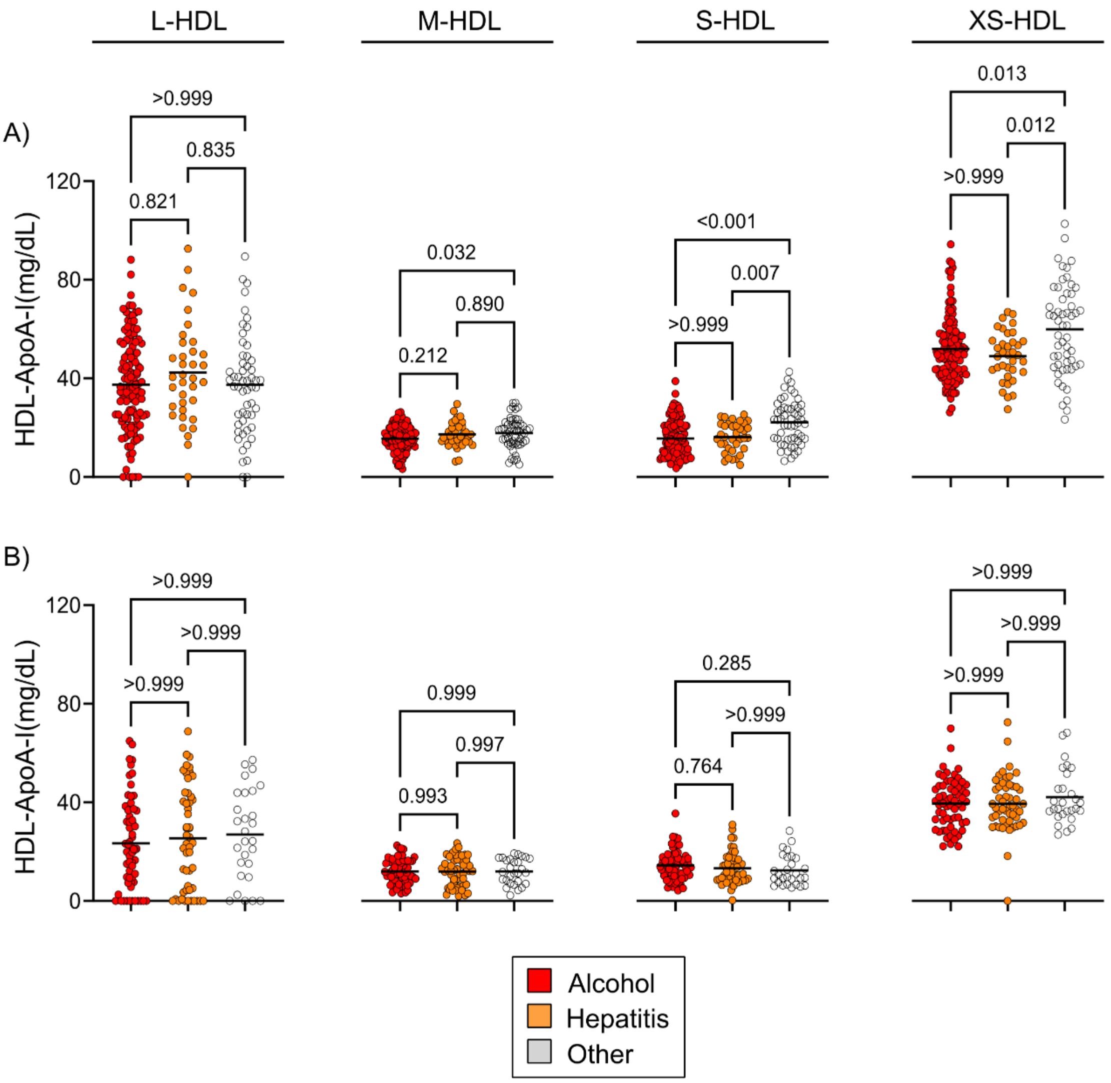

Figure S1. HDL Subclass Concentrations by Cirrhosis Etiology. Concentrations of HDL-ApoA-I subclasses (L, M, S, XS) in (**A**) compensated and (**B**) decompensated cirrhosis, stratified by alcohol-related, viral, and other etiologies. Statistical significance was determined using the Kruskal-Wallis test with Dunn's multiple comparisons post-hoc analysis. ApoA-I, apolipoprotein A-I; HDL, high-density lipoprotein.

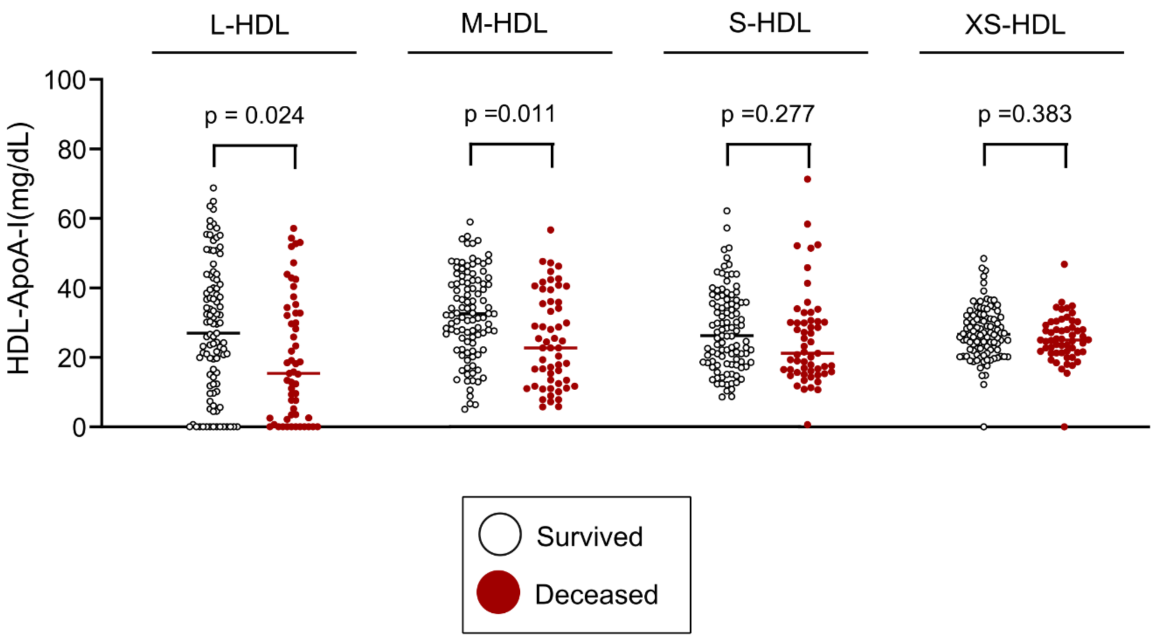

Figure S2. HDL-ApoA-I Subclass Concentrations and 12-Month Survival. Comparison of HDL-ApoA-I subclass concentrations (L, M, S, XS) between decompensated patients who survived 12 months and those who died. Statistical differences were assessed using unpaired t-tests or Mann-Whitney U tests, as appropriate. ApoA-I, apolipoprotein A-I; HDL, high-density lipoprotein.

Table S1. To assess the impact of inflammation, we incorporated logarithmic C-reactive protein (CRP) levels into the multivariable Cox Regression Model (Table 2). We analyzed 12-month mortality for compensated patients and 90-day mortality for decompensated patients. Hazard Ratios (HRs) with 95% Confidence Intervals (CIs) were calculated per 1 standard deviation increase in HDL subclass levels, adjusting for age, sex, MELD score, and log CRP. Significant findings ($p < 0.05$) are indicated in bold. Abbreviations: ApoA-I, apolipoprotein A-I; CRP, C-reactive protein; HDL, high-density lipoprotein; HR, hazard ratio; L, large; M, medium; S, small; XS, extra small.

	**Compensated**		**Decompensated**	
Parameter	HR (95 % CI) Per 1 SD	*p*-value	HR (95 % CI) Per 1 SD	*p*-value
Total HDL-ApoA-I	0.74 (0.29-1.91)	0.541	0.65 (0.33-1.27)	0.209
L-HDL-ApoA-I	0.89 (0.38-2.06)	0.780	0.84 (0.49-1.46)	0.538
M-HDL-ApoA-I	0.17 (0.04-0.71)	**0.015**	0.64 (0.33-1.22)	0.171
S-HDL-ApoA-I	0.04 (0.01-0.27)	**0.001**	0.95 (0.42-2.11)	0.891
XS-HDL-ApoA-I	0.55 (0.20-1.52)	0.246	0.33 (0.17-0.66)	**0.001**
